# Oxidative stress and abnormal cholesterol metabolism in patients with post-cardiac arrest syndrome

**DOI:** 10.3164/jcbn.17-30

**Published:** 2017-07-28

**Authors:** Midori Nagase, Atsushi Sakurai, Atsunori Sugita, Nozomi Matsumoto, Airi Kubo, Yusuke Miyazaki, Kosaku Kinoshita, Yorihiro Yamamoto

**Affiliations:** 1 School of Bioscience and Biotechnology, Tokyo University of Technology, 1404-1 Katakura-cho, Hachioji, Tokyo 192-0982, Japan; 2 Division of Emergency and Critical Care Medicine, Department of Acute Medicine, Nihon University School of Medicine, 30-1 Oyaguchi Kamimachi, Itabashi-ku, Tokyo 173-8610, Japan

**Keywords:** coenzyme Q10, cholesterol metabolism, plasma free fatty acids, therapeutic hypothermia, prosaposin

## Abstract

Patients with post-cardiac arrest syndrome (PCAS) suffer from whole body ischemia/reperfusion injury similar to that experienced by newborn babies. Increased oxidative stress was confirmed in PCAS patients (*n* = 40) at the time of hospitalization by a significant increase in the percentage of the oxidized form of coenzyme Q10 in total coenzyme Q10 compared to age-matched healthy controls (*n* = 55). Tissue oxidative damage in patients was suggested by the significant increase in plasma levels of free fatty acids (FFA) and the significant decrease in polyunsaturated fatty acid contents in total FFA. A greater decrease in free cholesterol (FC) compared to cholesterol esters (CE) was observed. Therefore, the FC/CE ratio significantly increased, suggesting deficiency of lecithin-cholesterol acyltransferase secreted from the liver. Time course changes of the above parameters were compared among 6 groups of patients divided according to outcome severity. Rapid declines of FC and CE were observed in patients who died within a day, while levels remained unchanged in patients discharged in a week. These data suggest that liver function is one of the key factors determining the survival of patients. Interestingly, therapeutic hypothermia treatment enhanced the increment of plasma ratio of coenzyme Q10 to total cholesterol at the end of rewarming.

## Introduction

Ischemia/reperfusion is an important trigger of reactive oxygen species (ROS) formation.^(1–3)^ As direct evidence, Aki *et al.*^(4)^ and Ono *et al.*^(5)^ observed the continuous formation of superoxides in the rat jugular vein after forebrain ischemia and subsequent reperfusion promoted its production. Newborn babies suffer from whole body ischemia/reperfusion injury since their oxygen tensions change from 2–8% to 21% at birth.^(6)^ We previously demonstrated a significant increase in oxidative stress by measuring plasma antioxidants at 0, 1, 3 and 5 days after birth.^(7)^ Plasma levels of the most sensitive antioxidant, ascorbic acid, decreased daily to equilibrium values at 3 and 5 days.^(7)^ Percentages of the oxidized form of coenzyme Q10 (%CoQ10) in total coenzyme Q10, another blood marker of oxidative stress, in infants (25–31%) were significantly higher than those in healthy young adults (4.5%).^(7)^ We also measured plasma free fatty acids (FFA) and their composition as markers of tissue oxidative damage.^(7)^ FFA levels were highest at day 1 and decreased rapidly thereafter, whereas the content of oxidatively vulnerable polyunsaturated fatty acids (%PUFA) in total FFA was lowest at day 1 and then increased.^(7)^

Patients with post-cardiac arrest syndrome (PCAS) also suffer from whole body ischemia/reperfusion. Therefore, their oxidative stress is likely severe. However, few papers describe a decrease of antioxidants and an increase of oxidation products from lipids and proteins. In this paper, we evaluated oxidative stress in patients with PCAS by measuring plasma markers of oxidative stress in the circulation (antioxidants) and tissues (FFA and their composition). We also measured plasma levels of free cholesterol (FC) and cholesterol esters (CE). Their time course changes (0, 6 and 24 h, and 2 and 7 days after hospitalization) were compared among 6 groups of patients divided according to outcome severity as follows: died within a day; died within a week; died within a month; hospitalized for more than 2 weeks; hospitalized for less than 2 weeks; and discharged in a week. We will discuss important factors determining the survival of patients.

The survival rate to hospital discharge from PCAS in Japan was only 7.9% in 2014.^(3)^ To increase the survival rate, we treated about 70% of patients with therapeutic hypothermia (TH). Surprisingly, TH treatment enhanced the increase in plasma ratio of total coenzyme Q10 (TQ10) to total cholesterol (TC) at the end of rewarming. This implies that coenzyme Q10 was introduced to blood circulation by a lipoprotein-independent pathway not previously discussed.

## Subjects and Methods

### Study design

The present study was carried out in the Division of Emergency and Critical Care Medicine, Department of Acute Medicine, Nihon University School of Medicine during the period from 29 November 2005 to 4 August 2015. The study protocols were approved by the Ethical Committee of Nihon University School of Medicine, and patient samples were obtained in accordance with the Helsinki Declaration of 1964, as revised in 2001. Forty subjects (27 males aged 66.0 ± 15.8 years (mean ± SD) and 13 females aged 63.4 ± 20.8 years) were enrolled. The causes of PCAS were acute myocardial infarction (*n* = 10), ventricular fibrillation (5), suffocation (5), congestive heart failure (2), hyperkalemia (2), complete atrioventricular block (1), sick sinus syndrome (1), coronary spastic angina (1), chronic obstructive pulmonary disease (1), diffuse interstitial fibrosing pneumonia (1), pulmonary embolism (1), necrotizing fasciitis (1), sepsis (1), acute pancreatitis (1), diabetic ketoacidosis (1), gastric ulcer bleeding (1), subarachnoid hemorrhage (1), double outlet right ventricle (1), and unknown (3).

Patients were treated with conventional resuscitation methods and, if possible, TH treatment (34°C for 24 h and gradual rewarming to 36°C for 24 h) was introduced. In 32 of 40 cases, TH treatment was applied; however, in 5 cases treatment was not completed because of unstable blood pressure (4 cases) and low temperature caused by infection (1 case) (Table 1). TH treatment was not applied to 8 cases.

Heparinized plasma was collected when patients were hospitalized and at 6 and 24 h, and 2 and 7 days, and stored at −80°C until analysis.

### Analytical procedure

Plasma levels of vitamin E (VE), ubiquinol-10, ubiquinone-10, FC, and CE were determined as previously described with some modifications.^(8)^ In brief, plasma was extracted with 19 volumes of 2-propanol and the extract was analyzed by HPLC using an analytical column (Supelcosil LC-8, 5 µm, 25 cm × 4.6 mm i.d.; Supelco Japan, Tokyo, Japan), a reduction column (RC-10-1; Irica, Kyoto, Japan) and an amperometric electrochemical detector (Model Σ985; Irica) with an oxidation potential of +600 mV (vs Ag/AgCl) on a glass carbon electrode. The mobile phase consisted of 50 mM sodium perchlorate in methanol/2-propanol (9/1, v/v), delivered at a flow rate of 0.8 ml/min.

Plasma levels of ascorbic acid (VC), uric acid (UA) and unconjugated bilirubin (BR) were determined by HPLC on a bonded-phase aminopropylsilyl column (Supelcosil LC-NH_2_, 5 µm, 25 cm × 4.6 mm i.d.; Supelco Japan) with UV/VIS detection (265 nm for 0–15 min and 460 nm for 15–22 min) as described previously.^(9)^

Plasma FFA were derivatized with monodansylcadaverine for analysis by HPLC.^(10)^ Briefly, plasma samples (50 µl) were mixed with 200 µl of methanol and then centrifuged at 13,000 × *g* for 5 min. Aliquots (50 µl) of supernatants were mixed with 20 µl of methanol containing 25 µM tridecanoic acid (internal standard) and dried under a stream of nitrogen gas, and the residue was admixed with diethyl phosphorocyanidate (1 µl) and *N*,*N*-dimethylformamide (50 µl) containing monodansylcadaverine (2 mg/ml) and kept at room temperature in the dark for 20 min. A 5-µl sample was injected onto an octadecylsilyl column (3 µm, 3.3 cm × 4.6 mm i.d.; Supelco Japan) and a pKb-100 column (5 µm, 25 cm × 4.6 mm i.d.; Supelco Japan) connected in tandem. The FFA components were measured by fluorescence detection (Model 821-FP; Japan Spectroscopic, Tokyo, Japan) with excitation at 320 nm and emission at 520 nm. The mobile phase consisted of acetonitrile/methanol/water (17.5/65.0/17.5, v/v/v) delivered at a flow rate of 1.5 ml/min. The analytical columns were heated to 40°C.

Plasma levels of prosaposin (Psap), a coenzyme Q10 binding and transfer protein, were measured by a sandwich ELISA using monoclonal and polyclonal antibodies against human saposin B.^(11)^ Plasma was diluted 100 times with a phosphate-buffer saline containing 0.1% Triton X-100, 1 g/L NaN_3_, 10 g/L BSA, and 1 mM EDTA. Purified saposin B was used as a standard.^(11)^

### Statistical analysis

Data presented are mean values and standard deviations. Statistical analysis was performed with a paired Student’s *t* test for two comparisons and one-way repeated measures ANOVA followed by the Tukey–Kramer multiple comparisons test. *P*<0.05 was considered statistically significant.

## Results and Discussion

### Oxidative stress in PCAS patients

Table 2 shows plasma levels of antioxidants and lipids in PCAS patients at the time of hospitalization and those in age-matched healthy controls. Age-matching is important for such comparison because serum levels of coenzyme Q10 and total cholesterol change with age.^(12)^ A significant increase in %CoQ10 was observed in PCAS patients compared to healthy controls, indicating that the redox balance of coenzyme Q10 shifted to the oxidized form, confirming increased oxidative stress in the blood of PCAS patients. A significant increase in plasma FFA levels suggests that considerable tissue damage occurred in PCAS patients. This damage is likely oxidative because a significant decrease in %PUFA was observed. Tissue damage results in the decomposition of DNA and the conversion of purines to UA. This is consistent with the observed significant increase in plasma UA. In contrast, plasma levels of VC, VE, and TQ10 in PCAS patients were similar to healthy controls. The significant decrease in BR is notable, since decreased BR is recognized as a risk factor for coronary artery disease.^(13,14)^

It is noteworthy that infants also exhibited high %CoQ10 (25–31%) at birth.^(7)^ Plasma FFA level were the highest at day 1, decreasing thereafter.^(7)^ Whereas, plasma %PUFA were the lowest at day 1 and then increased,^(7)^ and plasma levels of UA were the highest at day 1.^(7)^ The similarity in data between patients with PCAS and newborns is reasonable, since both are exposed to ischemia/reperfusion-induced oxidative stress.

Plasma levels of FC, CE, and TC were significantly lower than those in age-matched healthy controls. Moreover, the FC/CE ratio was significantly greater than that in controls, indicating decreased activity of lecithin-cholesterol acyltransferase (LCAT), which catalyzes the conversion of FC to CE.^(15,16)^ Since LCAT is secreted from the liver, an increased FC/CE ratio suggests impairment of liver function.^(15,16)^

### Time course changes in plasma antioxidants and cholesterol

**Fig. 1:**
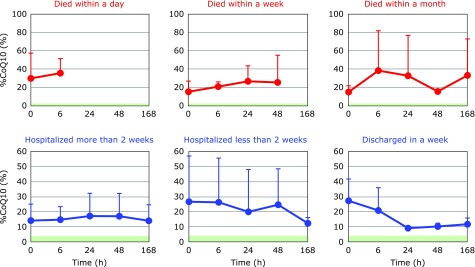
Time course of changes in the percentage of the oxidized form of coenzyme Q10 in TQ10 (%CoQ10) after hospitalization. Patients were divided into six groups according to outcome. Average %CoQ10 in age-matched healthy controls was 3.9 ± 1.3 (± SD, *n* = 55); this range is shaded in green.

**Fig. 2:**
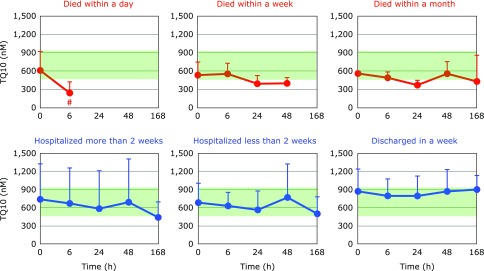
Time course of changes in plasma total coenzyme Q10 (TQ10) after hospitalization. Patients were divided into six groups according to outcome. The average TQ10 level in age-matched healthy controls was 710 ± 206 µM (± SD, *n* = 55); this range is shaded in green. ^#^*p*<0.05, significant differences compared to values at 0 h as determined by a paired Student’s *t* test.

**Fig. 3:**
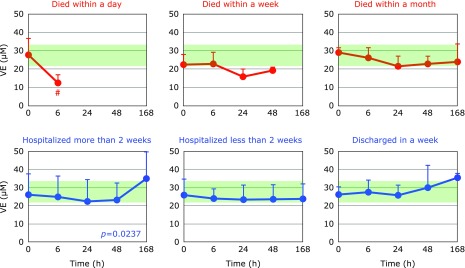
Time course of changes in plasma vitamin E (VE) after hospitalization. Patients were divided into six groups according to outcome. The average VE level in age-matched healthy controls was 28.5 ± 7.3 µM (± SD, *n* = 55); this range is shaded in green. ^#^*p*<0.05, significant differences compared to values at 0 h as determined by a paired Student’s *t* test. *P* values are indicated when one-way repeated ANOVA analysis was significant.

**Fig. 4:**
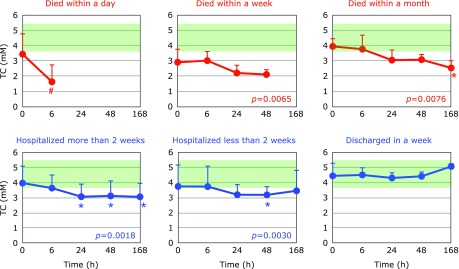
Time course of changes in plasma total cholesterol (TC) after hospitalization. Patients were divided into six groups according to outcome. The average TC level in age-matched healthy controls was 4.72 ± 0.94 mM (± SD, *n* = 55); this range is shaded in green. ^#^*p*<0.05, significant differences compared to values at 0 h as determined by a paired a Student’s *t* test. *P* values are indicated when one-way repeated ANOVA analysis was significant. *p<0.05, significant differences compared to values at 0 h as determined by the Tukey–Kramer multiple comparisons test.

**Fig. 5:**
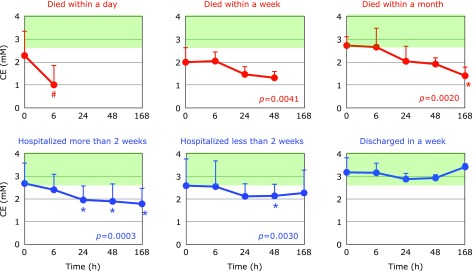
Time course of changes in plasma cholesterol esters (CE) after hospitalization. Patients were divided into six groups according to outcome. The average CE level in age-matched healthy controls was 3.36 ± 0.72 mM (± SD, *n* = 55); this range is shaded in green. ^#^*p*<0.05, significant differences compared to values at 0 h as determined by a paired Student’s *t* test. *P* values are indicated when one-way repeated ANOVA analysis was significant. *p<0.05, significant differences compared to values at 0 h as determined by the Tukey–Kramer multiple comparisons test.

**Fig. 6:**
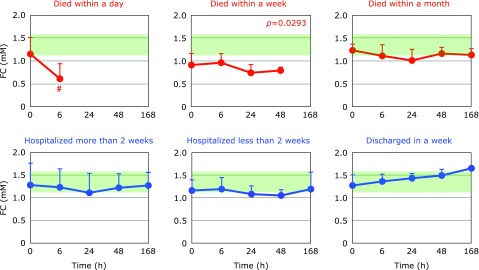
Time course of changes in plasma free cholesterol (FC) after hospitalization. Patients were divided into six groups according to outcome. The average FC level in age-matched healthy controls was 1.37 ± 0.25 mM (± SD, *n* = 55); this range is shaded in green. ^#^*p*<0.05, significant differences compared to values at 0 h as determined by a paired Student’s *t* test. *P* values are indicated when one-way repeated ANOVA analysis was significant. *p<0.05, significant differences compared to values at 0 h as determined by the Tukey–Kramer multiple comparisons test.

**Fig. 7:**
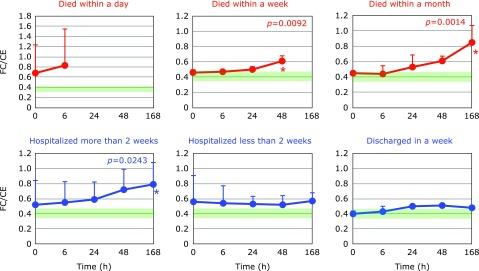
Time course of changes in plasma ratio of free cholesterol to cholesterol esters (FC/CE) after hospitalization. Patients were divided into six groups according to outcome. The average FC/CE ratio in age-matched healthy controls was 0.41 ± 0.05 (± SD, *n* = 55); this range is shaded in green. ^#^*p*<0.05, significant differences compared to values at 0 h as determined by a paired Student’s *t* test. *P* values are shown when one-way repeated ANOVA analysis was significant. *p<0.05, significant differences compared to values at 0 h as determined by the Tukey–Kramer multiple comparisons test.

**Fig. 8:**
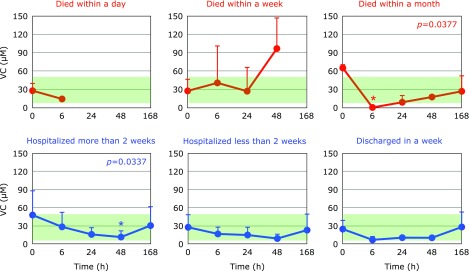
Time course of changes in plasma ascorbic acid (VC) after hospitalization. Patients were divided into six groups according to outcome. The average VC level in age-matched healthy controls was 31.1 ± 21.0 µM (± SD, *n* = 55); this range is shaded in green. *P* values are shown when one-way repeated ANOVA analysis was significant. *p<0.05, significant differences compared to values at 0 h as determined by the Tukey–Kramer multiple comparisons test.

**Fig. 9:**
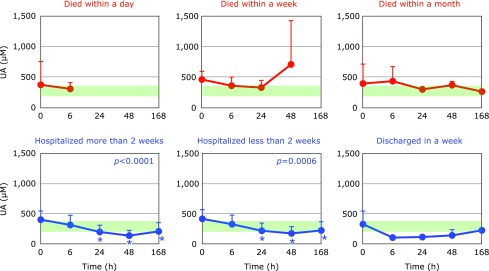
Time course of changes in plasma uric acid (UA) after hospitalization. Patients were divided into six groups according to outcome. The average UA level in age-matched healthy controls was 317 ± 86 µM (± SD, *n* = 55); this range is shaded in green. ^#^*p*<0.05, significant differences compared to values at 0 h as determined by a paired Student’s *t* test. *P* values are shown when one-way repeated ANOVA analysis was significant. *p<0.05, significant differences compared to values at 0 h as determined by the Tukey–Kramer multiple comparisons test.

Next, we examined time course (0–7 days after hospitalization) changes in plasma antioxidants and lipids as shown in Fig. 1–9. Patients with PCAS were divided into 6 groups according to outcome severity as follows: died within a day (6 cases); died within a week (6); died within a month (4); hospitalized for more than 2 weeks (11); hospitalized for less than 2 weeks (10); and discharged in a week (3). We expect that comparisons among these 6 groups could reveal important factors in determining the survival of patients.

Fig. 1 shows the time course changes of the oxidative stress marker %CoQ10 in the circulation. %CoQ10 values increased with time in patients who went on to die. In contrast, %CoQ10 values decreased in patients who were discharged within one or two weeks. These results clearly show that control of oxidative stress in blood circulation is important for patient survival.

Fig. 2 and 3 show the time course changes in lipid-soluble antioxidants, TQ10 and VE, respectively. Surprisingly, more than 50% of TQ10 and VE levels were lost over 6 h in patients who died within a day. However, these declines may not be associated with oxidative stress, since ~50% of TC also disappeared in 6 h (Fig. 4). Lipoprotein secretion from the liver and other organs was obviously impaired in patients who died within a day. In contrast, plasma levels of TC, TQ10, and VE in patients who were discharged in a week remained constant and within normal ranges (Fig. 2–4).

Fig. 5 and 6 show the time course changes in plasma CE and FC, respectively. It is apparent that the declines of CE were more profound than those of FC. This can be seen more easily in the FC/CE ratios shown in Fig. 7. The FC/CE ratios remained constant and within a normal range in PCAS patients who were discharged in a week. On the other hand, the FC/CE ratios were extremely high in PCAS patients who died within a day. This ratio increased with time in PCAS patients who died and those hospitalized for more than 2 weeks. Since the FC/CE ratio is determined by LCAT activity, which converts FC to CE, and LCAT is secreted with HDL from the liver, a high FC/CE ratio indicates some impairment of liver function. Supplementation with coenzyme Q10 could be one approach to preserve liver function, since improved FC/CE ratios were observed in patients with fibromyalgia upon supplementation.^(17)^

Fig. 8 and 9 show the time course changes in water-soluble antioxidants, VC and UA, respectively. Plasma levels of VC can be increased when many tissue cells are disrupted, because tissue cells contain mM levels of VC while plasma contains levels of ~30 µM. Such increases in VC levels were observed at 48 h in PCAS patients who died within a week (Fig. 8). It is reasonable that UA levels also increased at this point, because tissue disruption results in the conversion of purines to UA (Fig. 9). However, a significant decrease in UA was observed in PCAS patients who were hospitalized for more than a week (Fig. 9). This may due to the formation of peroxynitrite since UA is a specific inhibitor of peroxynitrite.^(18,19)^

**Fig. 10:**
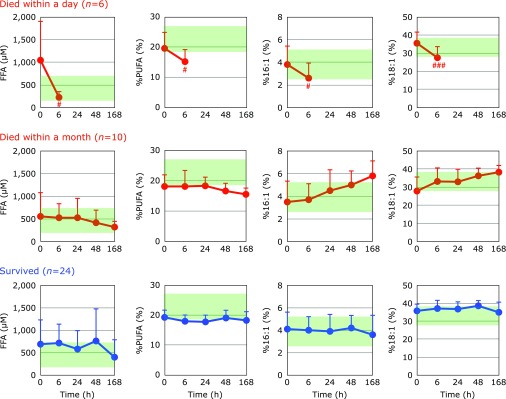
Time course of changes in plasma free fatty acids (FFA), the percentage of polyunsaturated fatty acids in total FFA (%PUFA), the percentage of palmitoleic acid in total FFA (%16:1), and the percentage of oleic acid in total FFA (%18:1) after hospitalization. Patients were divided into three groups according to outcome. The average FFA level, %PFA, %16:1, and %18:1 in age-matched healthy controls were 457 ± 288 µM, 23.6 ± 4.6, 3.9 ± 1.4, and 34.4 ± 5.1, respectively (± SD, *n* = 55); these ranges are shaded in green. ^#^*p*<0.05 and ^###^*p*<0.001, significant differences compared to values at 0 h as determined by a paired Student’s *t* test.

We have employed plasma FFA and the content of oxidatively vulnerable PUFA in total FFA as markers of tissue oxidative damage.^(20)^ It is common that stearoyl-CoA desaturase is activated to compensate for the loss of PUFA; therefore, the percentages of palmitoleic acid and oleic acid in total FFA (%16:1 and %18:1, respectively) are also appropriate markers of tissue oxidative damage.^(20)^ Fig. 10 shows the time course changes in FFA, %PUFA, %16:1 and %18:1. Here, we divided patients into 3 groups: those who died within a day (6 cases), died within a month (10), and survived (26). No significant changes were observed in patients who survived. In contrast, %PUFA decreased, and %16:1 and %18:1 increased with time in patients who died within a month, indicating ongoing tissue oxidative damage in these patients. However, all parameters significantly decreased at 6 h in patients who died within a day, suggesting that the above lipid preserving reactions had ceased in those patients.

### Effect of therapeutic hypothermia treatment

**Fig. 11:**
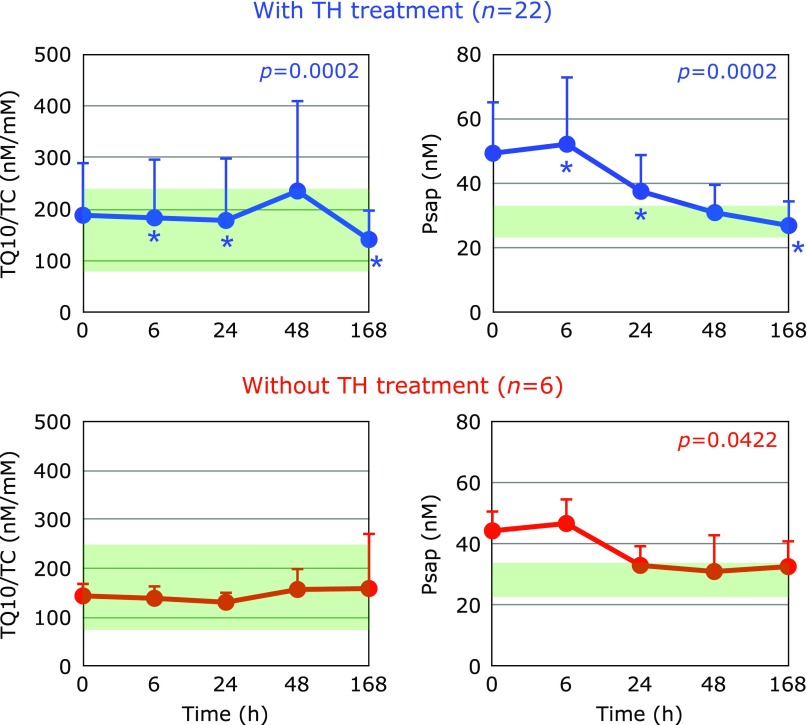
Time course of changes in ratio of plasma total coenzyme Q10 to total cholesterol (TQ10/TC) and plasma prosaposin (Psap) after hospitalization. Patients were divided into groups with (*n* = 22) and without (*n* = 6) therapeutic hypothermia (TH) treatment. The average Psap level and TQ10/TC ratio in age-matched healthy controls were 27.2 ± 5.8 nM (± SD, *n* = 80) and 180 ± 86 nM/mM (± SD, *n* = 55), respectively; these ranges are shaded in green. *P* values are shown when one-way repeated ANOVA analysis was significant. *p<0.05, significant differences compared to values at 48 h as determined by the Tukey–Kramer multiple comparisons test.

The efficacy of TH treatment against PCAS is generally acknowledged in Japan.^(21,22)^ TH treatment is employed to reduce the formation of oxygen radicals. In fact, TH treatment decreased superoxide formation in an animal model of ischemia/reperfusion injury.^(23)^ Moreover, we observed that TH treatment induced a significant increase in plasma TQ10/TC at the end of rewarming (48 h) compared to the 24 h values and this ratio was significantly decreased at day 7 (Fig. 11). This was not the case in the absence of TH treatment (Fig. 11). Since the ratios of TQ10/TC were increased, this indicates that the increment is not lipoprotein-dependent. Thus, we focused on the levels of Psap as a coenzyme Q10 binding and transfer protein in plasma.^(11,24,25)^

Psap is a multifunctional glycoprotein present in all organs as the lysosomal precursor of four small sphingolipid activator proteins, known as saposin A, B, C, and D, and also exists as a secreted protein, which has been found in various bodily fluids such as serum, milk, and seminal fluid.^(26,27)^ Notably, plasma levels of Psap in PCAS patients at the time of hospitalization were 47.3 ± 15.1 (± SD, *n* = 36), significantly higher than in age-matched healthy controls (27.2 ± 5.8, *n* = 80) (*p*<0.001). Since plasma Psap levels decreased with time (Fig. 11), the elevation of Psap levels preceded the alterations in TQ10/TC ratio. Although the detailed mechanism requires further study, it is of interest that the human body appears to require coenzyme Q10 under critical conditions such as PCAS.

## Conclusion

In summary, increased oxidative stress was confirmed in PCAS patients at the time of hospitalization by the significant increases in plasma %CoQ10 and FFA, and the significant decrease in %PUFA. Impairment of liver function was suggested by an increase in FC/CE ratio. A time course study revealed that this ratio is one of the key factors in determining the survival of patients. TH treatment enhanced increases in the plasma ratio of TQ10 to TC at the end of rewarming via a lipoprotein-independent pathway.

## Figures and Tables

**Table 1:** Outcomes of the 40 PCAS patients and therapeutic hypothermia treatment

Outcome	Died/Hospitalized	Therapeutic hypothermia treatment			Total
		Performed	Ceased	Untreated	
Died	<1 day	2	1	3	6
Died	<1 week	3	2	1	6
Died	<1 month	2	0	2	4
Survived	>2 week	9	2	0	11
Survived	1–2 week	10	0	0	10
Survived	1 week	1	0	2	3
Total		27	5	8	40

**Table 2:** Levels of plasma antioxidants and lipids in patients with PCAS at the time of hospitalization as compared to age-matched healthy controls (average ± SD)

	PCAS	Normal control	*p*
*n*	40	55	
Male/Female	27/13	38/17	
Age	65.2 ± 17.4	60.1 ± 9.3	
VC (µM)	36.5 ± 28.9	31.1 ± 21.0	
UA (µM)	406 ± 191	317 ± 86	<0.05
BR (µM)	4.1 ± 3.7	6.9 ± 3.6	<0.01
VE (µM)	26.0 ± 8.8	28.5 ± 7.3	
TQ10 (nM)	667 ± 393	710 ± 206	
%CoQ10	20.1 ± 21.0	3.9 ± 1.3	<0.05
FFA (µM)	709 ± 608	457 ± 288	<0.001
%PUFA	19.1 ± 3.5	23.6 ± 4.6	<0.001
%16:1	3.9 ± 1.7	3.9 ± 1.4	
%18:1	33.8 ± 6.6	34.4 ± 5.1	
FC (mM)	1.17 ± 0.35	1.37 ± 0.25	<0.001
CE (mM)	2.54 ± 0.95	3.36 ± 0.72	<0.001
TC (mM)	3.71 ± 1.20	4.72 ± 0.94	<0.001
FC/CE	0.53 ± 0.33	0.41 ± 0.05	<0.05

*P* values were determined using a Student’s *t* test. VC, ascorbic acid; UA, uric acid; BR, unconjugated bilirubin; VE, vitamin E; TQ10, total coenzyme Q10; %CoQ10, ratio of oxidized form of coenzyme Q10 to TQ10; FFA, free fatty acids; %PUFA, ratio of polyunsaturated fatty acids to total free fatty acids; %16:1, ratio of palmitoleic acid to total FFA; %18:1, ratio of oleic acid to total FFA; FC, free cholesterol; CE, cholesterol esters; TC, total cholesterol.

## Abbreviations

BR: unconjugated bilirubin
CE: cholesterol esters
%CoQ10: percentage of oxidized form of coenzyme Q10 in TQ10
FC: free cholesterol
FFA: free fatty acids
LCAT: lecithin-cholesterol acyltransferase
PCAS: post-cardiac arrest syndrome
Psap: prosaposin
%PUFA: percentage of polyunsaturated fatty acids in total FFA
TH: therapeutic hypothermia
TC: total cholesterol
TQ10: total coenzyme Q10
UA: uric acid
VC: ascorbic acid
%16:1: percentage of palmitoleic acid in total FFA
%18:1: percentage of oleic acid in total FFA

## References

[1] Warner DS, Sheng H, Batinić-Haberle I (2004). Oxidants, antioxidants and the ischemic brain. J Exp Biol.

[2] Bagheri F, Khori V, Alizadeh AM, Khalighfard S, Khodayari S, Khodayari H (2016). Reactive oxygen species-mediated cardiac-reperfusion injury: mechanisms and therapies. Life Sci.

[3] Uchino H, Ogihara Y, Fukui H (2016). Brain injury following cardiac arrest: pathophysiology for neurocritical care. J Intensive Care.

[4] Aki HS, Fujita M, Yamashita S (2009). Elevation of jugular venous superoxide anion radical is associated with early inflammation, oxidative stress, and endothelial injury in forebrain ischemia-reperfusion rats. Brain Res.

[5] Ono T, Tsuruta R, Fujita M (2009). Xanthine oxidase is one of the major sources of superoxide anion radicals in blood after reperfusion in rats with forebrain ischemia/reperfusion. Brain Res.

[6] Fischer B, Bavister BD (1993). Oxygen tension in the oviduct and uterus of rhesus monkeys, hamsters and rabbits. J Reprod Fertil.

[7] Hara K, Yamashita S, Fujisawa A, Ishiwa S, Ogawa T, Yamamoto Y (1999). Oxidative stress in newborn infants with and without asphyxia as measured by plasma antioxidants and free fatty acids. Biochem Biophys Res Commun.

[8] Yamashita S, Yamamoto Y (1997). Simultaneous detection of ubiquinol and ubiquinone in human plasma as a marker of oxidative stress. Anal Biochem.

[9] Yamamoto Y, Ames BN (1987). Detection of lipid hydroperoxides and hydrogen peroxide at picomole levels by an HPLC and isoluminol chemiluminescence assay. Free Radic Biol Med.

[10] Yamamoto Y, Nagata Y, Katsurada M, Sato S, Ohori Y (1996). Changes in rat plasma-free fatty acids composition under oxidative stress induced by carbon tetrachloride: decrease of polyunsaturated fatty acids and increase of palmitoleic acid. Redox Rep.

[11] Jin G, Kubo H, Kashiba M (2008). Saposin B is a human coenzyme Q10-binding/transfer protein. J Clin Biochem Nutr.

[12] Niklowitz P, Onur S, Fischer A (2016). Coenzyme Q10 serum concentration and redox status in European adults: influence of age, sex, and lipoprotein concentration. J Clin Biochem Nutr.

[13] Schwertner HA, Jackson WG, Tolan G (1994). Association of low serum concentration of bilirubin with increased risk of coronary artery disease. Clin Chem.

[14] Kunutsor SK, Bakker SJ, Gansevoort RT, Chowdhury R, Dullaart RP (2015). Circulating total bilirubin and risk of incident cardiovascular disease in the general population. Arterioscler Thromb Vasc Biol.

[15] Florén CH, Chen CH, Franzén J, Albers JJ (1987). Lecithin: cholesterol acyltransferase in liver disease. Scand J Clin Lab Invest.

[16] Yamamoto Y, Yamashita S, Fujisawa A, Kokura S, Yoshikawa T (1998). Oxidative stress in patients with hepatitis, cirrhosis, and hepatoma evaluated by plasma antioxidants. Biochem Biophys Res Commun.

[17] Miyamae T, Seki M, Naga T (2013). Increased oxidative stress and coenzyme Q10 deficiency in juvenile fibromyalgia: amelioration of hypercholesterolemia and fatigue by ubiquinol-10 supplementation. Redox Rep.

[18] Pacher P, Beckman JS, Liaudet L (2007). Nitric oxide and peroxynitrite in health and disease. Physiol Rev.

[19] Nagase M, Yamamoto Y, Miyazaki Y, Yoshino H (2016). Increased oxidative stress in patients with amyotrophic lateral sclerosis and the effect of edaravone administration. Redox Rep.

[20] Yamamoto Y (2017). Plasma marker of tissue oxidative damage and edaravone as a scavenger drug against peroxyl radicals and peroxynitrite. J Clin Biochem Nutr.

[21] Komatsu T, Kinoshita K, Sakurai A (2014). Shorter time until return of spontaneous circulation is the only independent factor for a good neurological outcome in patients with postcardiac arrest syndrome. Emerg Med J.

[22] Kaneko T, Kasaoka S, Nakahara T (2015). Effectiveness of lower target temperature therapeutic hypothermia in post-cardiac arrest syndrome patients with a resuscitation interval of ≤30 min. J Intensive Care.

[23] Koda Y, Tsuruta R, Fujita M (2010). Moderate hypothermia suppresses jugular venous superoxide anion radical, oxidative stress, early inflammation, and endothelial injury in forebrain ischemia/reperfusion rats. Brain Res.

[24] Kashiba M, Oizumi M, Suzuki M (2014). Prosaposin regulates coenzyme Q10 levels in HepG2 cells, especially those in mitochondria. J Clin Biochem Nutr.

[25] Kashiba M, Terashima M, Sagawa T, Yoshimura T, Yamamoto Y (2017). Prosaposin knockdown in Caco-2 cells decreases cellular levels of coenzyme Q10 and ATP, and results in the loss of tight junction barriers. J Clin Biochem Nutr.

[26] O'Brien JS, Kishimoto Y (1991). Saposin proteins: structure, function, and role in human lysosomal storage disorders. FASEB J.

[27] Kishimoto Y, Hiraiwa M, O'Brien JS (1992). Saposins: structure, function, distribution, and molecular genetics. J Lipid Res.

